# Report of Three Cases of AKI Following Weight-Based Gentamicin Prophylaxis for IPP Implantation: Potential Concerns for Patients with Preexisting Conditions

**DOI:** 10.1155/2018/3479202

**Published:** 2018-12-04

**Authors:** Robert H. Moore, Uzoma A. Anele, Sarah C. Krzastek, Adam P. Klausner, J. Tyler Roseman

**Affiliations:** ^1^Virginia Commonwealth University School of Medicine, 1201 E. Marshall Street, Richmond, VA 23298, USA; ^2^Division of Urology, Virginia Commonwealth University Medical Center, West Hospital, 7th Floor, 1200 E. Broad Street, Richmond, VA 23298-0118, USA; ^3^Division of Urology, Hunter Holmes McGuire VA Medical Center, 1201 Broad Rock Blvd., Richmond, VA 23249, USA

## Abstract

Despite the known nephrotoxicity of gentamicin, in 2008 the American Urological Association published guidelines recommending single high-dose weight-based gentamicin prophylaxis of 5 mg/kg for procedures involving urologic prostheses. These guidelines are based on the theoretical renal safety and improved antimicrobial activity of a single large dose of gentamicin. However, the risk of nephrotoxicity after weight-based gentamicin prophylaxis specifically in penile prosthetic surgery has never been established with evidence-based studies. This is of special concern in light of the known high rates of preexisting conditions in this specific population. Therefore, in order to expose potential safety issues, we present three cases of postoperative acute kidney injury following weight-based gentamicin prophylaxis after implantation of inflatable penile prostheses.

## 1. Introduction

Aminoglycosides such as gentamicin have long been used in urologic prosthetic surgery and have recently regained popularity due to their low cost and relative efficacy [1]. Although nephrotoxicity is a well-documented risk with these agents, a growing body of evidence has shown aminoglycoside nephrotoxicity to be more dependent on duration of dose, rather than peak serum levels [1]. In 2008 the American Urological Association (AUA) incorporated this information into their recommendation for single high-dose preoperative intravenous (IV) gentamicin prophylaxis of 5 mg/kg for urologic prosthetic implantation [2]. Our institution adopted this guidance (along with 2-3 mg/kg ideal body weight [or adjusted body weight if obese] in cases of decreased renal function), coupled with 10-20 mg/kg IV vancomycin, prior to induction of anesthesia. Despite these guidelines, there is a paucity of evidence regarding the renal safety of weight-based gentamicin during urologic prosthetic surgery.

A recent retrospective study comparing weight-based to nonweight-based gentamicin dosing in inflatable penile prosthesis (IPP) implantation found no difference in the rate of postoperative acute kidney injury (AKI) [3]. However, the study was limited by the lack of any postoperative measures of nephrotoxicity and only evaluated rates of readmission for AKI. Interestingly, the majority of gentamicin-associated cases of AKI are subclinical stage-1 insults, making readmission rates an inadequate outcome measure [4]. In addition, there are no specific recommendations regarding dose adjustment for patients with preexisting conditions such as chronic kidney disease (CKD) or diabetes mellitus (DM) which are highly prevalent in individuals with medication-refractory erectile dysfunction. Therefore, we present three cases of postoperative AKI following weight-based gentamicin prophylaxis for IPP implantation in order to highlight potential safety issues with this type of antibiotic prophylaxis particularly in individuals with preexisting conditions that might predispose to AKI.

## 2. Case Presentations

*Case 1*. A 64-year-old male with a medical history significant for hyperlipidemia, hypertension, DM type 2, CKD, and prostate cancer treated with radiotherapy presented to the urology service for 3-piece IPP placement due to erectile dysfunction refractory to medical management. Preoperative anesthesia assessment 14 days prior revealed a serum creatinine of 1.41 mg/dL and an estimated glomerular filtration rate (eGFR) of 65.08 mL/min/1.73 m^2^. On the day of surgery, the patient received IV gentamicin 160 mg (2.46 mg/kg ideal body weight) and IV vancomycin 1000 mg (8.96 mg/kg) 11 minutes prior to first incision. IPP (3-piece Coloplast Titan® Touch, Minneapolis MN, USA) placement was performed without complication following intraoperative placement of a Foley catheter. A total of 1,807 mL IV normal saline was administered intraoperatively and systolic blood pressure remained above 90 mmHg throughout the procedure, of which the total operative duration was 147 minutes. A Jackson-Pratt (JP) drain was placed in the right hemiscrotum, and the catheter was maintained.

Postoperatively, the patient was restarted on his home medications. On the 1st postoperative day, the patient was afebrile without nausea or vomiting, and with adequate urine output. Drain outputs were minimal, prompting removal. Notably, the patient's serum creatinine was found to have risen to 2.92 mg/dL (eGFR 28.09 mL/min/1.73 m^2^). Repeat serum creatinine that afternoon showed a continued rise to 3.04 mg/dL (eGFR 26.82 mL/min/1.73 m^2^), consistent with a stage-2 AKI by Kidney Disease: Improving Global Outcomes (KDIGO) criteria. The nephrology service was consulted and recommended continuing IV hydration. On the 2nd postoperative day, urine output was again adequate, and serum creatinine was found to be stable at 3.02 mg/dL (eGFR 27.02 mL/min/1.73 m^2^). The patient was discharged and on postoperative follow-up 44 days later, his renal function was found to have largely improved to a serum creatinine of 1.68 mg/dL (eGFR 53.17 mL/min/1.73 m^2^). 

*Case*  *2*. A 65-year-old male with a history of hypertension, DM type 2, and refractory erectile dysfunction underwent IPP implant. During preoperative anesthesia assessment 21 days prior, he demonstrated baseline renal function with a serum creatinine of 1.02 mg/dL and eGFR of 94.27 mL/min/1.73 m^2^. Intraoperatively, he received IV gentamicin 300 mg (3.73 mg/kg ideal body weight) and IV vancomycin 1000 mg (8.73 mg/kg) within 20 minutes prior to first incision. The IPP (3-piece Coloplast Titan® Touch, Minneapolis MN, USA) was inserted without complication during the total operative duration of 153 minutes. The patient received 1,800 mL IV normal saline intraoperatively.

On the 1st postoperative day, the patient remained clinically well and his Foley catheter and JP drains were removed. However, his serum creatinine was discovered to increase to 2.67 mg/dL (eGFR 31.05 mL/min/1.73 m^2^), consistent with a stage-2 AKI. Repeat serum creatinine that evening demonstrated a continued rise to 3.92 mg/dL (eGFR 19.94 31.05 mL/min/1.73 m^2^), a stage-3 AKI. On the 2nd postoperative day, the patient's serum creatinine rose further to 5.95 mg/dL (eGFR 12.32 mL/min/1.73 m^2^) at which point he became transiently oliguric. The nephrology service was consulted and hemodialysis was initiated. By the 4th postoperative day, his serum creatinine increased to a peak of 7.11 mg/dL (eGFR 10.03 mL/min/1.73 m^2^). His urine output eventually began to improve and by the 7th postoperative day, his serum creatinine improved to 4.98 mg/dL (eGFR 15.12 mL/min/1.73 m^2^). He was then discharged with instructions to follow-up in nephrology clinic. At postoperative follow-up 19 days later, the patient's serum creatinine had continued trending down to 1.53 mg/dL (eGFR 59.04 mL/min/1.73 m^2^), and 6 months postoperatively the patient's renal function further improved to a serum creatinine 1.22 mg/dL (eGFR 76.43 mL/min/1.73 m^2^), notably still above his original baseline. 

*Case*  *3*. A 65-year-old male with a medical history significant for obesity, hypertension, CKD, DM type-2, and erectile dysfunction managed with IPP placement 12 years prior presented for evaluation of his intermittently malfunctioning device. The decision was made to proceed with device revision. Preoperative serum creatinine was found to be 1.55 mg/dL (eGFR of 58.16 mL/min/1.73 m^2^) 14 days prior to surgery. He received IV gentamicin 230 mg (3.07 mg/kg ideal body weight) and IV vancomycin 2000 mg (17.12 mg/kg) within 30 minutes prior to first incision. The previous IPP (3-piece AMS 700™ Boston, Massachusetts, USA) device was successfully explanted (with the exception of the reservoir) and replaced (3-piece Coloplast Titan® One Touch Release, Minneapolis MN, USA) without complication. A total of 1,000 mL IV normal saline and 500 mL IV 5% dextrose in water (D5W) was administered intraoperatively during the 147 min procedure.

He began convalescing appropriately postoperatively and his drains were removed on day 1; however, he developed a stage-1 AKI as demonstrated by an increase of serum creatinine to 2.09 mg/dL (eGFR 41.19 mL/min/1.73 m^2^). On the 2nd postoperative day, the serum creatinine was found to have stabilized at 1.98 mg/dL (eGFR 43.85 mL/min/1.73 m^2^) and he was subsequently discharged. On postoperative follow-up 24 days later, the patient's renal function normalized to a serum creatinine of 1.54 mg/dL (eGFR 58.42 mL/min/1.73 m^2^).

## 3. Discussion

This study identified three cases of AKI after weight-based gentamicin for IPP implantation, all occurring in individuals with preexisting conditions that could predispose to renal injury (Table 1). These episodes of AKI occurred despite our use of lower gentamicin doses in this population, even though this type of dose adjustment is not discussed in guidelines. Gentamicin maintains antimicrobial activity through its concentration-dependent bacterial activity which is positively correlated with peak concentrations and outcomes [1]. The higher the peak to minimum inhibitory concentration (MIC) ratio, the greater the extent and rate of bacterial eradication. Interestingly, clinical studies have suggested that high-dosing (i.e., weight-based) may result in decreased nephrotoxicity compared to traditional doses through decreased renal tubular accumulation of gentamicin. The major mechanism of gentamicin-induced nephrotoxicity is mediated by active transport of the drug into proximal convoluted tubule cells: a process which is saturable at even standard therapeutic serum concentrations [1]. Therefore, the nephrotoxicity is more dependent on duration of administration, rather than the strength of the individual preoperative dose now recommended by the AUA [1, 2]. Despite this theoretical renal safety, the patients in each case developed AKI in the absence of any clear prerenal etiologies such as hypotension or volume depletion. Several risk factors shared among these cases may have contributed to an aminoglycoside induced AKI, including preexisting conditions (e.g., hypertension CKD and DM) and coadministration with another nephrotoxic agent, vancomycin [1]. The antibacterial effect of vancomycin is based on time-dependent exposure as measured by the ratio of the 24-hour area under the concentration-time curve (AUC) to the MIC. Although trough levels are typically used as a surrogate for this measure, supratherapeutic concentrations may result in nephrotoxicity [5]. However, the influence of vancomycin on AKI could not be fully assessed due to the lack of measured serum levels.

AKI occurred despite the reduced gentamicin dose of 2-3 mg/kg ideal body weight (or adjusted body weight if obese). Studies suggest that the majority of gentamicin-associated AKIs are transient and many patients, such as* Case*  *3*, may ultimately return to baseline renal function [4]. However, transient AKI can worsen baseline renal function, as seen in* Cases*  *1 and*  2, and even significantly increase the risk of future end-stage renal disease [6]. These cases highlight potential safety concerns regarding weight-based gentamicin prophylaxis in certain patient populations undergoing urologic prosthetic surgical procedures and the need for larger scale, evidence-based investigations examining nephrotoxic effects of this recommended antibiotic dosing.

## Figures and Tables

**Table 1:** Patient characteristics.

Patient	Age (years)	Comorbidities	Gentamicin Dose (mg/kg)	Vancomycin Dose (mg/kg)	Pre-op Serum Cr [eGFR]	Post-op Peak Serum Cr [eGFR]	Follow-up Serum Cr [eGFR]
Case 1	64	HTN, DM-2, CKD, HLD	2.46	8.96	1.41 [65.08]	3.04 [26.82]	1.68 [53.17]
Case 2	65	HTN, DM-2	3.73	8.73	1.02 [94.27]	7.11 [10.03]	1.22 [76.43]
Case 3	65	Obesity, HTN, CKD, DM-2	3.07	17.12	1.55 [58.16]	2.09 [41.19]	1.54 [58.42]

AKI = acute kidney injury, HTN = hypertension, DM-2 = diabetes mellitus type-2, CKD = chronic kidney disease, HLD = hyperlipidemia, IPP = inflatable penile prosthesis, Cr = creatinine (in mg/dL), and eGFR = estimated glomerular filtration rate (in mL/min/1.73 m^2^).
